# Research on the Precise Differentiation of Pathological Subtypes of Non-Small Cell Lung Cancer Based on ^18^F-FDG PET/CT Radiomics Features

**DOI:** 10.3390/cancers17203311

**Published:** 2025-10-14

**Authors:** Wenbo Li, Linjun Ju, Shuxian Zhang, Zheng Chen, Yue Li, Yuyue Feng, Yuting Xiang, Tingxiu Xiang, Zhongjun Wu, Hua Pang

**Affiliations:** 1 Department of Nuclear Medicine, The First Affiliated Hospital of Chongqing Medical University, Chongqing 400016, China; 204385@hospital.cqmu.edu.cn (W.L.); 205199@hospital.cqmu.edu.cn (L.J.);; 2 Department of Pathology, College of Basic Medicine, Chongqing Medical University, Chongqing 400016, China; 3 Chongqing Key Laboratory for the Mechanism and Intervention of Cancer Metastasis, Chongqing University Cancer Hospital, Chongqing University, Chongqing 400030, China; xiangtx@cqmu.edu.cn; 4 Department of Hepatobiliary Surgery, The First Affiliated Hospital of Chongqing Medical University, Chongqing 400016, China

**Keywords:** PET/CT, NSCLC, nomograph, radiomics, pathological subtype

## Abstract

**Simple Summary:**

Using 18F-FDG PET/CT radiomics features within and around tumors, combined with clinical characteristics, to accurately distinguish between different pathological subtypes of non-small-cell lung cancer (NSCLC). Radiomics feature extraction was performed on 18F-FDG PET/CT images of primary tumors and surrounding tumor regions using LIFE-x (5.2.0). Multivariate logistic regression analysis was used to construct a nomogram for distinguishing between lung adenocarcinoma (LUAD) and lung squamous cell carcinoma (LUSC). A nomogram model constructed by combining radiomic features extracted from 18F-FDG PET/CT images of tumors and surrounding tissues with clinical features can accurately distinguish between LUAD and LUSC.

**Abstract:**

**Objectives**: Employing 18F-FDG PET/CT radiomic properties both within and surrounding tumors, in conjunction with clinical attributes, to precisely differentiate among several pathological subtypes of non-small-cell lung cancer (NSCLC). **Approaches**: The study comprised 222 patients who received 18F-FDG PET/CT scans from January 2015 to December 2020 and were later diagnosed with NSCLC, encompassing 169 cases of lung adenocarcinoma (LUAD) and 53 cases of lung squamous cell carcinoma (LUSC). They were arbitrarily allocated into a training group and a validation group in a 7:3 ratio. Radiomics feature extraction was conducted on 18F-FDG PET/CT images of primary tumors and adjacent tumor regions with LIFE-x (5.2.0). A multivariate logistic regression analysis was employed to develop a nomogram for differentiating lung adenocarcinoma (LUAD) from lung squamous cell carcinoma (LUSC). The clinical efficacy of each model was assessed and contrasted utilizing accuracy (Acc), sensitivity (Sen), specificity (Spe), receiver operating characteristic curve (ROC), calibration curve, and decision curve analysis (DCA). **Outcomes**: The nomogram model that integrates 18F-FDG PET/CT radiomics features with clinical characteristics showed superior efficacy in differentiating adenocarcinoma from squamous cell carcinoma in NSCLC patients, surpassing models based only on PET or CT radiomics. The validation set exhibited an Area under curve (AUC) of 0.880, an Acc of 0.929, a Sen of 0.808, and a Spe of 0.962. This model exhibits the most superior overall performance in DCA. **Conclusions**: A nomogram model integrating radiomic features derived from 18F-FDG PET/CT images of tumors and adjacent tissues with clinical characteristics can effectively differentiate between LUAD and LUSC.

## 1. Introduction

Non-small cell lung cancer (NSCLC) constitutes the primary subtype of lung cancer, accounting for approximately 80–85% of all lung cancer cases [1,2,3,4], Lung adenocarcinoma (LUAD) and lung squamous cell carcinoma (LUSC) are the two main pathological forms. Pathological subtypes of NSCLC demonstrate notable variations in biological behavior, treatment response, and prognosis. LUAD exhibits greater sensitivity to targeted therapy, whereas LUSC predominantly depends on chemotherapy or immunotherapy [5,6,7,8,9,10]. In order to create individualized treatment programs and enhance patient outcomes, it is crucial to precisely identify the histological subtypes of NSCLC prior to surgery.

Pathological biopsy is recognized as the definitive method for diagnosing subtypes of NSCLC. This method is invasive and may result in diagnostic inaccuracies due to sampling errors, with limited applicability for patients with advanced or inoperable disease [11,12,13,14]. Imaging studies serve as essential non-invasive assessment tools in the diagnosis of lung cancer [15,16,17,18]. 8F-fluorodeoxyglucose positron emission tomography/computed tomography (18F-FDG PET/CT) is a non-invasive imaging modality that identifies abnormal glucose metabolism in tumor cells, thereby offering metabolic insights into tumors without invasive procedures. It offers substantial support for the early detection, staging, and efficacy evaluation of tumors and has been widely employed in the diagnosis, staging, and efficacy assessment of lung cancer. Specifically, 18F-FDG PET/CT has demonstrated significant sensitivity and specificity in the diagnosis of NSCLC [19,20,21,22,23,24].

Radiomics has recently emerged as a significant field, focusing on the extraction of quantitative features from medical images, including texture, morphological, and metabolic features, utilizing high-throughput methods. Combined with machine learning algorithms, radiomics can uncover potential biological information that is not visible to the naked eye, providing new quantitative evidence for tumor subtype differentiation and prognosis prediction [16,18]. Prior research indicates that radiomic features derived from CT or PET imaging may effectively differentiate between lung adenocarcinoma and squamous cell carcinoma [23,25,26,27]. The combined analysis of multi-modal PET/CT radiomics and its efficacy in differentiating NSCLC subtypes necessitates additional investigation.

This study seeks to develop a machine learning model utilizing 18F-FDG PET/CT radiomics features to evaluate its effectiveness in accurately differentiating major pathological subtypes of NSCLC, specifically LUAD and LUSC. The objective is to offer clinicians a non-invasive and efficient diagnostic tool for subtype classification, thereby aiding in personalized treatment decisions for NSCLC.

## 2. Materials and Methods

### 2.1. Study Design and Inclusion Criteria

**Figure 1:**
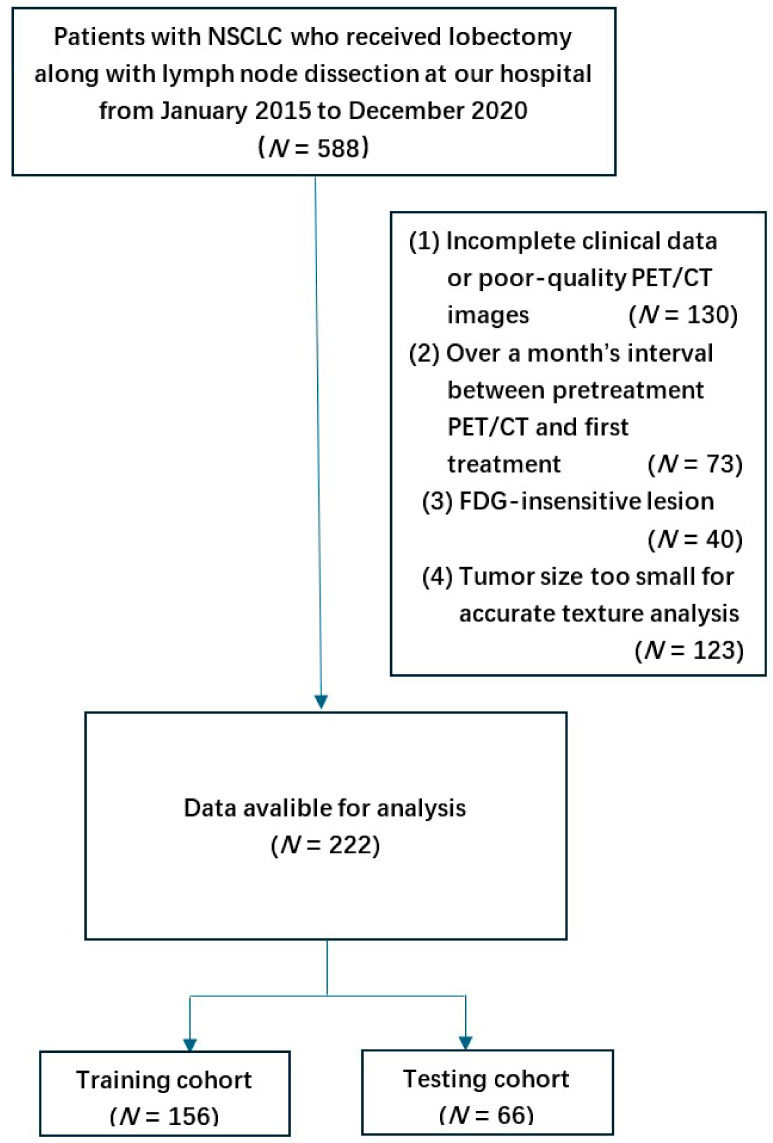
The flowchart of inclusion and exclusion criteria of patients.

This is a retrospective, observational study sanctioned by the ethics committee of the First Affiliated Hospital of Chongqing Medical University (Ethics number: 2021-341). This study comprised 588 patients with NSCLC who received lobectomy along with lymph node dissection at our hospital from January 2015 to December 2020. The inclusion and exclusion criteria for patients are as follows Figure 1: the inclusion criteria were pathologically confirmed NSCLC and preoperative 18F-FDG-PET/CT examination data; the exclusion criteria were Incomplete clinical data or poor-quality PET/CT images (*N* = 130), over a month’s interval between pretreatment PET/CT and first treatment (*N* = 73), FDG-insensitive lesions (*N* = 40), and tumor size too small for accurate texture analysis (*N* = 123).

A total of 222 patients were enrolled and subsequently assigned to training and testing cohorts in a 7:3 ratio through random division. A comprehensive review of the medical records for all patients was conducted, focusing on various parameters such as age, sex, smoking history, histologic typing, TNM stage, lesion location, lesion size, treatment modality, and serum levels of tumor markers, which include cytokeratin 19 fragment antigen 21-1 (CYFRA 21-1), pro-gastrin-releasing peptide (Pro-GRP), squamous cell carcinoma antigen (SCCA), carcinoembryonic antigen (CEA), and neuron-specific enolase (NSE).

### 2.2. PET/CT Image Acquisition and Tumor Segmentation

All patients underwent a fasting period of no less than 6 h prior to the examination, ensuring that their fasting blood glucose levels were at or below 11.1 mmol/L. Patients received intravenous injections of 18F-FDG visualizer at a calculated dosage ranging from 3.70 MBq/kg. Patients were observed in a state of rest for a duration of 60 min before undergoing PET/CT imaging, which covered a scanning range extending from the top of the head to the mid-upper thigh. The patients underwent scanning with a Philips Gemini 64 PET/CT scanner (Philips Medical Systems, Amsterdam, The Netherlands), utilizing specific CT scanning parameters: voltage set at 120 kV, current at 100 mA, and a layer thickness of 4.0 mm. The parameters for PET scanning included a layer thickness of 4.0 mm and a duration of 1 min per bed. The scans underwent computer attenuation and were reconstructed through iterative methods to achieve maximum-intensity projections and fused images.

Two types of regions of interest (ROIs) were annotated from PET/CT images, encompassing both intra-tumoral and peri-tumoral areas. Manually delineated polygonal ROIs were employed to segment intra-tumoral regions on selected fused PET/CT image slices. Intra-tumoral regions: Two nuclear medicine physicians with 10 years’ experience in interpreting pulmonary PET/CT independently delineated intra-tumoral regions using LIFE-x (5.2.0). Where multiple tumor lesions were present, only the largest lesion was selected for segmentation. Differences in their delineation results were then carefully reconciled through consensus. Following manual lesion segmentation, the tumor region of interest (ROI) was defined as the area retained by applying a 40% threshold based on the lesion’s maximum standardized uptake value (SUVmax).

Peri-tumoral ROI: On PET/CT image slices with tumor-internal regions of interest (ROIs), morphological dilation and erosion techniques in 3D Slicer software version 5.0.3 (www.slicer.org) were employed to define the tumor periphery boundary at a radial distance of 5 mm from the primary tumor. The area remaining after subtracting the tumor-internal ROI constituted the tumor-peripheral ROI. Physicians manually excluded imaging data from normal tissues such as bronchi, major vessels, and the mediastinum.

### 2.3. Radiomic Feature Extraction

Radiomic features were gathered from the primary and peritumoral regions of interest (ROIs) utilizing LIFE-x version 5.2.0. LIFE-x the software is only capable of texture analysis for ROIs with volumes greater than 64 voxels, so 123 patients did not meet the requirements to extract texture features. Ultimately, 48 CT features and 50 ROI features were extracted Table 1.

### 2.4. Feature Selection

Min-max deviation normalisation was performed on all radiomic features, and the missing values were interpolated to means. In the training cohort, the features were downscaled using the least absolute selection and shrinkage operator (LASSO), and the regularisation parameter λ was selected using ten-fold cross-validation to determine the final selection. Next, we selected six groups of features: PET primary features are PET radiomics features extracted from the tumor-internal ROI and selected corresponding to λ.1se of Lasso regression, whilst PET peri-tumoral features are PET radiomics features extracted from the peri-tumoral ROI selected corresponding to λ.1se of Lasso regression. There are two PET combined datasets, obtained by screening all PET radiomics features extracted from the tumor-internal ROI and the peri-tumoral ROI using the Lasso regression parameters λ.1se and λ.min, respectively. The CT combined dataset refer to all CT radiomics features extracted from the tumor-internal ROI and the peri-tumoral ROI, filtered according to the λ.min parameter of Lasso regression.

### 2.5. Establishment of the Models

Univariate analysis of clinical factors was conducted in both the training and testing cohorts to identify independent predictors and address between-group differences. Following modelling, the following sets of models were obtained:

PET primary model: A logistic regression model incorporating solely PET primary features corresponding to λ.1se of Lasso regression.

PET external model: A logistic regression model incorporating solely PET peritumoral features corresponding to λ.1se of Lasso regression.

PET plus models: A composite of primary lesion characteristics and peritumoral features on PET, corresponding to two logistic regression models with λ.1se and λ.min parameters in Lasso regression.

CT plus model: A logistic regression model incorporating both CT primary features and CT peritumoral features corresponding to λ.min of Lasso regression.

PET/CT plus model: A logistic regression model incorporating both PET/CT primary features and PET/CT peritumoral features corresponding to λ.min of Lasso regression.

PET/CT plus and clinical model: A logistic regression model utilizing the sum of PET/CT primary lesion characteristics and PET/CT tumor perivascular features corresponding to the Lasso regression λ.min threshold, alongside clinical factors significantly associated with pathological subtypes in the training cohort (*p* < 0.001).

### 2.6. Model Performances

Models were evaluated in a testing cohort. We considered two scenarios. First, considering the added value of PET peritumoral radiomics, we compared the following three models: PET primary, PET external, and PET plus. The model demonstrating the highest predictive performance was chosen for advancement to subsequent comparative analyses. Next, we compared the following four models: CT plus, PET plus, PET/CT plus, and PET/CT plus and clinical models. We estimated the accuracy, specificity, sensitivity, and the AUC of each. We also constructed calibration curves and conducted decision curve analyses (DCAs) to evaluate models further. Finally, we used the best model to construct the nomogram.

**Figure 2:**
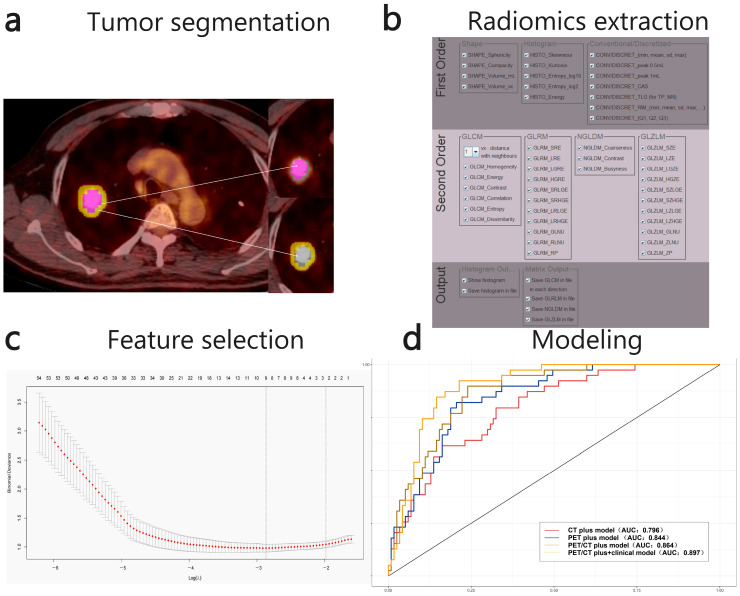
The workflow included tumor segmentation, radiomic feature extraction, feature selection, statistical analysis and modeling. Tumor segmentation, where in the red region is designated as the tumor-internal region of interest (ROI) and the yellow region as the peri-tumoral ROI (**a**); Radiomics feature extraction (**b**); Feature selection (**c**); Statistical analysis and modelling, specifically the ROC curves of the PET Primary model, PET External model and PET plus model in the training cohort (**d**).

Statistical analysis was performed using the R software. (version 4.3.0, http://www.R-project.org). Comparisons of continuous variables were done using the *t*-testing or Wilcox rank-sum test, and categorical data were analyzed using the chi-square or Fisher’s exact testing depending on the data. The process of extracting feature parameters for radiomics is shown in Figure 2.

To quantify uncertainty and evaluate over-fitting beyond the single 7:3 split, we performed two additional internal-validation procedures on the entire cohort of 222 patients:1000-bootstrap resampling (sampling with replacement) to estimate 95% confidence intervals (CIs) for AUC, sensitivity and specificity; stratified 10-fold cross-validation repeated 10 times to assess partition stability. Bootstrap analyses were implemented with the ROC package (v-1.18.0) in R (v-4.3.1); cross-validation was carried out with the caret package. A difference between apparent AUC and cross-validation AUC < 0.02 was considered negligible over-fitting.

## 3. Results

### 3.1. Patient Clinical Characteristics

The research involved 222 patients with NSCLC, comprising 169 individuals with LUAD and 53 with LUSC. The dataset was randomized with a training to testing ratio of 7:3, comprising 156 cases in the training cohort (117 LUAD and 39 LUSC patients) and 66 cases in the testing cohort (52 LUAD and 14 LUSC patients). The comparison of clinical characteristics between the two groups revealed no statistically significant differences (*p* > 0.05). Table 2 presents the characteristics of all patients. Smoking history and CYFRA21-1 were significantly more prevalent in LUSC patients compared to LUAD patients, irrespective of the training cohort (*p* < 0.001) or the testing cohort (*p* = 0.002). These factors were also identified as clinically independent in distinguishing LUAD from LUSC.

### 3.2. Prediction as an Additional Value of PET Peritumoral Radiomics

**Figure 3:**
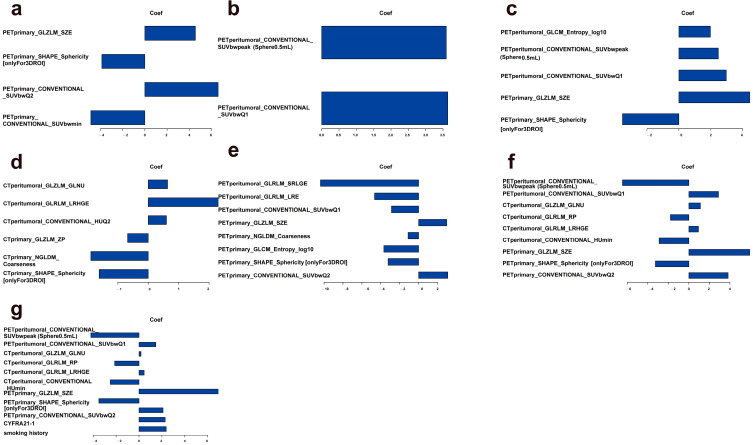
Retained radiomics features and corresponding coefficients of different models. models by constructed using the radiomics features corresponding to λ.1se in LASSO regression. PET Primary model (**a**); PET External model (**b**); PET plus model (**c**). models by constructed using the radiomics features corresponding to λ.min in LASSO regression: CT Plus model (**d**); PET plus model (**e**); PET/CT plus model (**f**); PET/CT and Clinical model (**g**).

**Figure 4:**
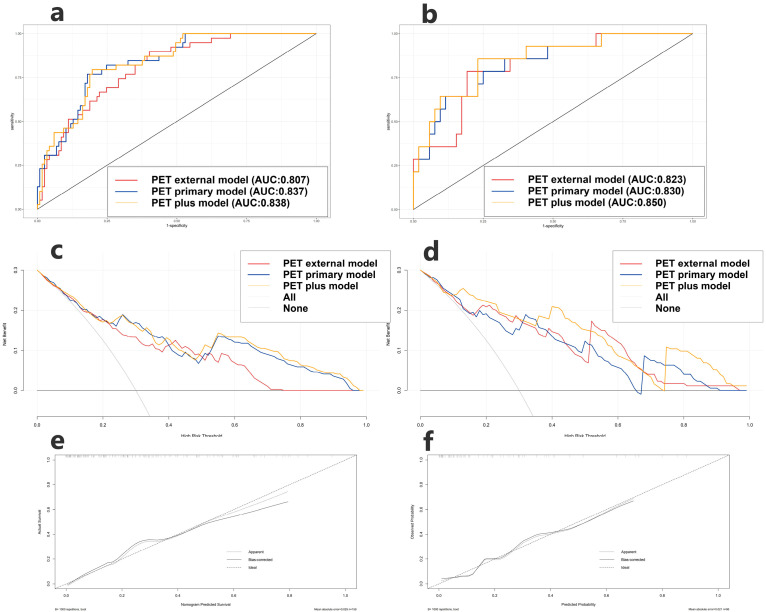
ROC of the created PET Primary model, PET External model and PET plus model in the training cohort (**a**) and testing cohort (**b**). Decision curve analysis of the created PET Primary model, PET External model and PET plus model in the training cohort (**c**) and testing cohort (**d**). Calibration curve of PET plus model displays in the training cohort (**e**) and testing cohort (**f**).

The radiomic features selected in the model and their corresponding logistic regression coefficients are shown in Figure 3. All models showed good predictive ability for distinguishing between LUAD and LUSC in the training and testing cohorts Figure 4 and Table 3. The PET plus model exhibited the highest AUC value across both cohorts. The rest of the findings pertain to the testing cohort only. The PET plus model had higher sensitivity and specificity vs. PET primary (sensitivity = 0.857 vs. 0.786, specificity = 0.769 vs. 0.750), and higher sensitivity vs. but slightly lower specificity vs. PET peritumoral (sensitivity = 0.857 vs. 0.786, specificity = 0.769 vs. 0.808). There was little difference in the accuracy among the three models. In DCA, PET plus model again had the highest overall best performance. In calibration curve, the prediction curve was similar to a diagonal line, indicating that its predictions had a good fit with the actual data. Therefore, this model outperformed all others.

Next, to further evaluate the added value of peritumoral radiomics, we computed the net reclassification improvement (NRI) and integrated discrimination improvement (IDI) in the testing cohort. The NRI for the PET plus model vs. the PET primary model was 0.409 (95%CI = 0.132-0.687, *p* = 0.004), and the IDI was 0.089 (95%CI = −0.005-0.184, *p* = 0.063). Therefore, the classification accuracy of the PET plus model was superior to that of the PET primary model.

### 3.3. Model Optimization

As seen in Figure 3, we constructed three models in the training cohort: the PET plus, the CT plus, and the PET/CT plus models. Furthermore, we explored the clinical characteristics substantially correlated with pathological subtypes in the training cohort: smoking history and CYFRA21-1 (*p* < 0.001), together with LASSO-selected PET/CT main tumor and peritumoral radiomics features.

**Figure 5:**
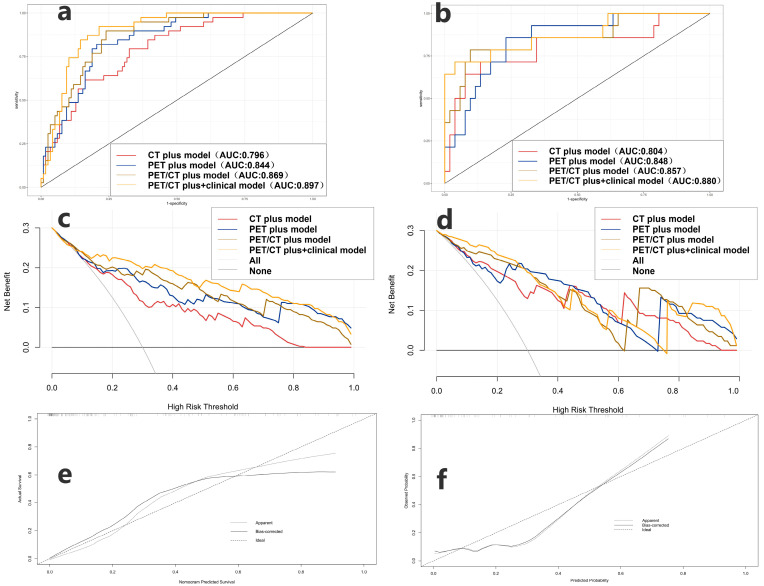
ROC of the created PET plus model, CT plus model, PET/CT plus model and PET/CT plus and clinical model in the training cohort (**a**) and testing cohort (**b**). Decision curve analysis of the created PET plus model, CT plus model, PET/CT plus model and PET/CT plus and clinical model in the training cohort (**c**) and testing cohort (**d**). Calibration curve of PET/CT plus and clinical model in the training cohort (**e**) and testing cohort (**f**).

All models in the training (AUC = 0.897, 0.869, 0.844, 0.796) and testing cohorts (AUC = 0.875, 0.857, 0.848, 0.804) performed well. (Figure 5 and Table 3). The PET/CT plus and clinical model exhibited the highest AUC value among the evaluated options. In the testing cohort, this model demonstrated the highest accuracy and specificity, although its sensitivity was marginally lower than that of the PET plus model (ACC = 0.909, Sen = 0.714, Spe = 0.962). The PET/CT plus and clinical model in DCA exhibited the highest overall net clinical benefit in the training cohort, with the calibration curve indicating strong correlations between predictions and actual observations in both cohorts. Consequently, the model exhibited optimal performance.

The PET/CT plus model demonstrated superior performance compared to both the CT plus model and the PET plus model in the testing cohort (Table 3) (AUC = 0.857, ACC = 0.864, Sen = 0.786, Spe = 0.907). The model demonstrated superior AUC, sensitivity, specificity, and accuracy compared to the CT plus model (AUC = 0.805, ACC = 0.818, Sen = 0.714, Spe = 0.865), as well as higher AUC, specificity, and accuracy (with marginally lower sensitivity) compared to the PET plus model (AUC = 0.805, ACC = 0.818, Sen = 0.714, Spe = 0.865).

### 3.4. Nomogram Construction of the PET/CT Plus and Clinical Model

**Figure 6:**
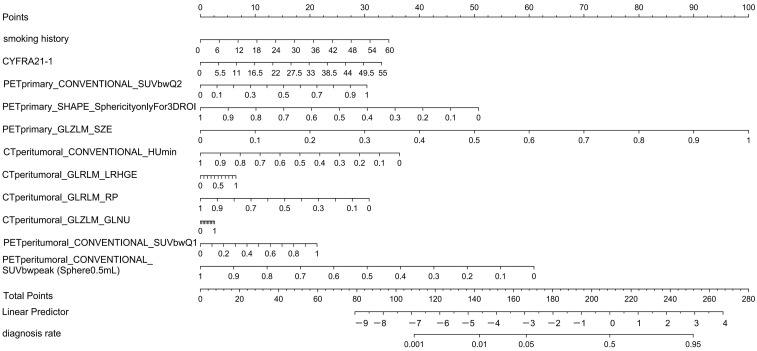
Nomograph constructed using PET/CT plus and clinical model.

Because the PET/CT plus and clinical model had the best predictive performances, we constructed a nomogram of this model based on the training cohort (Figure 6). The score for each patient based on the logistic regression coefficients is as follows:

Score = 2.379 × Smoking history + 2.288 × CYFRA211_1 + 2.102 × PETprimary_CON VENTIONAL_SUVbwQ2 − 3.511 × PETprimary_SHAPE_SphericityonlyFor3DROI + 6.916 × PETprimary_GLZLM_SZE − 2.513 × CTexternal_CONVENTIONAL_HUmin + 0.449 × CTexternal_GLRLM_LRHGE-2.130 × CTexternal_GLRLM_RP + 0.179 × CTexternal_GLZLM_GLNU + 1.472 × PETexternal_CONVENTIONAL_SUVbwQ1 − 4.211 × PETexternal_CONVENTIONAL_SUVbwpeakSphere0.5 mLvalueonlyforPETorNM.

Across the entire cohort (*N* = 222), following 1000-fold bootstrap resampling, the AUC value of this prediction map reached 0.826 (95% confidence interval 0.721–0.930). Repeated 10-fold cross-validation yielded an average AUC of 0.880, indicating negligible overfitting. No significant overfitting was observed in other models. Detailed metric summaries are presented in Table 4.

### 3.5. Precise Differentiation of Pathological Subtypes of Non-Small Cell Lung Cancer

**Figure 7:**
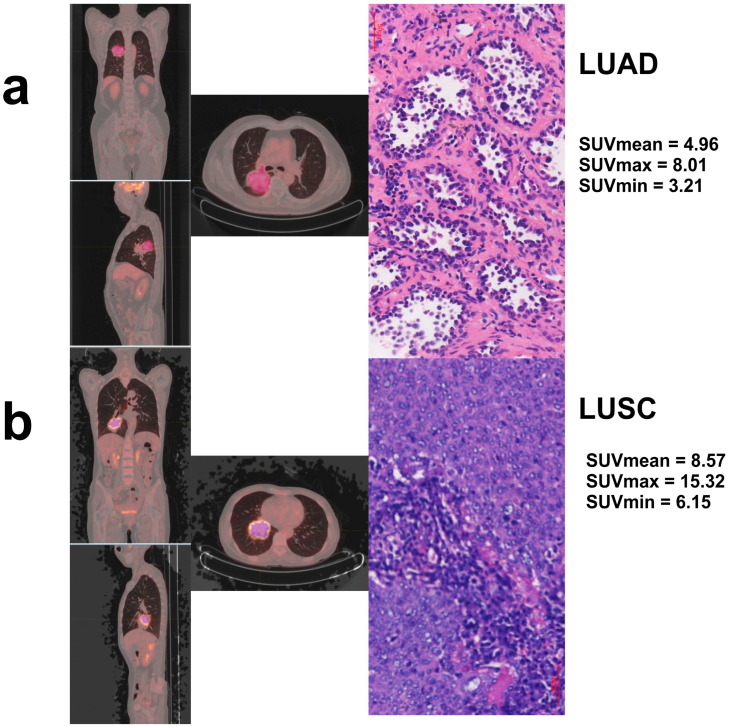
Typical cases of LUAD and LUSC. PET/CT image of a 68-year-old woman, the total score in the nomogram was 201.453, the probability of LUAD was 0.72, and the postoperative pathology confirmed LUAD (**a**). PET/CT image of a 54-year-old man, the total score in the nomogram was 53.902 and the probability of LUSC was 0.81, and the postoperative pathology confirmed LUSC (**b**).

The patient’s predicted score can be derived from the score associated with the predictive factors, as illustrated in Figure 6. A total score approaching 260 indicates a higher likelihood of LUAD; conversely, scores significantly lower suggest LUSC (Figure 7).

## 4. Discussion

The pathological subtypes of non-small cell lung cancer (NSCLC), notably Lung adenocarcinoma (LUAD) and Lung squamous cell carcinoma (LUSC), exhibit notable differences in their treatment options and prognostic outcomes. It is essential to precisely distinguish between the two in a clinical context [6,7]. Traditional methods mainly rely on tissue biopsy or postoperative pathology, but suffer from problems such as high invasiveness and sampling bias [14,28,29]. In recent years, radiomics have provided a new way for non-invasive pathology prediction by extracting deep-level features from medical images [30,31].

This study’s innovation is evident in its pioneering approach to predicting various pathological subtypes of NSCLC using PET/CT radiomics data from the tumor’s surroundings. It also systematically evaluates the predictive efficacy of CT, PET, and PET/CT radiomics data related to the tumor. The study revealed that a composite model integrating PET/CT radiomics with clinical features achieved the highest performance in differentiating LUAD from LUSC, evidenced by a validation set AUC of 0.880. This result significantly surpassed the performance of PET or CT radiomics used independently, thereby affirming the enhanced efficacy of the combined PET/CT model with clinical features. Peritumoral PET radiomics demonstrates incremental predictive value compared to primary tumor PET radiomics. Additionally, we developed a nomogram for the PET/CT combined clinical model, which can be used for individualized prediction.

This result is consistent with the recent trend of multimodal radiomics research. For instance, Ren et al. [32] developed a clinical-radiomics nomogram that achieved an AUC of 0.901 in the validation set, demonstrating the benefits of combining metabolic features with morphological data. The metabolic and textural characteristics of the peritumor region, a significant component of the tumor microenvironment, are likely to be intricately linked to the biological behavior of various pathological subtypes.

The peritumoral area, as the junction zone between the tumor and normal tissue, its radiomics features may contain key information about tumor invasiveness, angiogenesis and the immune microenvironment [33,34]. CT peritumoral radiomics studies indicate that the texture features of tumor margins correlate significantly with prognosis. Additionally, metabolic parameters from PET/CT, such as SUVmax, may provide insights into the metabolic heterogeneity of the peri-tumor region [32,35]. The high specificity of the PET/CT composite model in distinguishing adenocarcinoma from squamous carcinoma (0.962) in this study may be related to its simultaneous capture of peritumoral metabolic activity and morphological differences [26]. The benefits of PET/CT radiomics in forecasting the pathological subtypes of NSCLC are primarily evident in the following areas:

(1) Complementarity of metabolic and anatomical information: PET reflects the metabolic activities of tumors (such as 18F-FDG uptake), while CT provides the morphological features of tumors. The composite model of PET/CT imaging histology combines both metabolic and morphological information to provide a more comprehensive description of tumor heterogeneity [34,36]. For example, LUAD may exhibit high metabolic activity in the peri-tumor area, more pronounced invasiveness, and specific textural features (e.g., elevated entropy), whereas LUSC may exhibit lower metabolic activity, stronger fibrosis or necrosis, and a more regular texture. The predictive efficacy of the PET/CT composite model was significantly higher compared to PET or CT radiomics alone (AUC = 0.880 vs. PET or CT model alone), suggesting that the fusion of multimodal features can more effectively differentiate pathological subtypes.

(2) Synergistic effect of multimodal features: The combination of clinical features (e.g., smoking history, serum tumor markers) and radiomics significantly improves the predictive efficiency [18,37]. For example, Yin et al. [38] demonstrated that the integrated model combining clinical-metabolic features and PET/CT radiomics achieved an AUC of 0.870 in the validation set, outperforming the radiomics model independently. This synergy effect may stem from the complementarity between the biological background provided by clinical data (such as LUAD being more common in non-smokers) and radiomics features. In addition, the nomogram constructed in this study provides an intuitive tool for clinical decision-making by visualizing the risk scores, and the good fit of its calibration curve to the actual data further validates the reliability of the model.

(3) Biological significance of the peritumoral region: The peritumoral region is an important part of the tumor microenvironment, and its metabolic and textural features (e.g., SUV values, grey scale covariance matrix parameters), may be closely related to the invasiveness, metastatic potential and pathological type of the tumor. For example, the peritumoral region of adenocarcinomas may exhibit higher metabolic activity and more complex textural features, whereas the peritumoral region of squamous carcinomas may exhibit stronger fibrosis or necrosis. By extracting the radiomics features of the peritumor region, the biological behavior of the tumor can be more comprehensively reflected, thus enhancing the predictive efficacy [28,39,40].

Studies have shown that radiomics techniques utilizing 18F-FDG PET/CT can differentiate between LUSC and LUAD [28,29,32,38,41,42,43,44,45,46], with PET/CT models even outperforming CT models in diagnostic performance [32] (AUC = 0.910 vs. 0.710). Most studies have primarily focused on the tumor region, neglecting the tumor microenvironment. The region surrounding the primary tumor shows mild FDG uptake on PET images, but may actually harbor invasive cancer cells [19,47]. The tumor-infiltrated region may eventually experience a certain degree of hypoxia, which triggers angiogenesis through the secretion of related mediators by tumor cells, leading to changes in 18F-FDG uptake and providing additional information. Tumor peritumoral radiomics based on PET/CT or PET/MRI has been demonstrated to have predictive capability in assessing tumor invasiveness, lymph node metastasis, and prognosis [20,21,22,23,48].

Nonetheless, there is a lack of studies addressing the preoperative differential diagnosis of various pathological subtypes of NSCLC through the comparison of primary tumor PET/CT radiomics and peritumoral PET/CT radiomics, as well as the predictive capabilities of PET/CT in relation to CT peritumoral radiomics. This study is the first to incorporate the tumor peritumoral region from PET/CT into the analysis. By combining metabolic information, it further expands the application of radiomics in predicting NSCLC pathological subtypes. Its predictive performance (AUC 0.880) is comparable to that of Ren et al.’s PET/CT whole-tumor radiomics study (AUC 0.901), but it focuses more on the boundary zone characteristics of the tumor microenvironment.

Additionally, the definition of the peritumoral region (e.g., 15–30 mm beyond the tumor margin) aligns with the “dangerous margin” concept commonly used in prognostic studies, suggesting that this region may contain key signals of tumor invasion.

This study analysis the relationship between traditional clinical characteristics (age, sex, smoking history, tumor location, maximum diameter of tumor, lymph node metastasis, T stage, N stage, M stage, TNM stage, and levels of various serum tumor markers) and the pathological subtype of NSCLC. Patients with NSCLC, a smoking history, and higher levels of the CYFRA21-1 blood marker were more likely to develop SCC. This is consistent with previous studies.

This study has several limitations. Firstly, as a single-center retrospective investigation, all samples were sourced from our institution. Patient baseline characteristics may differ from those in other regions or hospitals of varying tiers, limiting the direct universality of findings to broader populations. Secondly, the relatively small single-center sample size (*N* = 222) may have reduced statistical power for detecting small effect sizes, necessitating cautious interpretation. Future studies should adopt a multicenter, prospective cohort design to expand sample size and enhance statistical power, whilst incorporating patients from diverse geographical locations and healthcare facilities to clarify the applicability of findings. Thirdly, the model was trained exclusively on lesions with volumes > 64 voxels (as segmented via LIFE-x), excluding 123 patients with smaller tumors. Consequently, its performance for small lesions remains unvalidated, and caution should be exercised when extrapolating predictions to such cases. Fourth, moving forward with PET, we shall establish the optimal boundary values for tumor peripheral regions through comparative studies. Fifth, the biological link between peri-tumoral radiomics in PET/CT and pathological subtypes of lung cancer remains incompletely understood. Future research may integrate molecular biology studies, such as in-depth exploration of correlations with EGFR mutations.

Finally, although the model achieved an AUC of 0.89, its sensitivity was only 0.714, meaning that 29 out of every 100 patients would be missed. This model cannot stand alone as an exclusion tool. We recommend combining the probability predictions with clinical assessment: for patients deemed inoperable or at high biopsy risk, clinicians may utilize this score for preliminary evaluation—a probability score ≥ 0.75 warrants a ‘strong recommendation for biopsy’; where the score is <0.75 but imaging is highly suspicious, multidisciplinary consultation should be initiated to consider liquid biopsy or further evaluation. Data monitoring revealed only one misclassification among 25 patients with risk scores exceeding 0.75. Future large-scale, multicenter prospective cohort studies are required to further calibrate thresholds and evaluate clinical outcomes.

The core value of this study lies in its pioneering assessment of the potential utility of peritumoral regions in PET/CT radiomics for determining pathological subtypes of non-small cell lung cancer, providing a new research perspective for this field.

## 5. Conclusions

Different pathological subtypes of NSCLC exhibit distinct radiomic features on 18F-FDG PET/CT. The nomogram model integrating radiomic features and clinical characteristics derived from FDG PET/CT effectively differentiates LUAD from LUSC.

## Figures and Tables

**Table 1:** PET/CT Radiomic.

	PET	CT
First order	CONVENTIONAL_SUVbwmin	CONVENTIONAL_HUmin
	CONVENTIONAL_SUVbwmean	CONVENTIONAL_HUmean
	CONVENTIONAL_SUVbwstd	CONVENTIONAL_HUstd
	CONVENTIONAL_SUVbwmax	CONVENTIONAL_HUmax
	CONVENTIONAL_SUVbwQ(1,2,3)	CONVENTIONAL_HUQ(1,2,3)
	CONVENTIONAL_SUVbwSkewness	CONVENTIONAL_HUSkewness
	CONVENTIONAL_SUVbwKurtosis	CONVENTIONAL_HUKurtosis
	CONVENTIONAL_SUVbwExcessKurtosis	CONVENTIONAL_HUExcessKurtosis
	CONVENTIONAL_SUVbwpeakSphere0.5mL(value only for PET or NM)	CONVENTIONAL_HUcalciumAgatston Score[onlyForCT]
	CONVENTIONAL_SUVbwpeakSphere1mL (value only for PET or NM)	
	CONVENTIONAL_TLG(mL) [onlyForPETorNM]	
	SHAPE_Volume(mL)	SHAPE_Volume(mL)
	SHAPE_Volume(vx)	SHAPE_Volume(vx)
	SHAPE_Sphericity[onlyFor3DROI])	SHAPE_Sphericity[onlyFor3DROI])
	SHAPE_Surface(mm^2^)[onlyFor3DROI]	SHAPE_Surface(mm^2^)[onlyFor3DROI]
	SHAPE_Compacity[onlyFor3DROI]	SHAPE_Compacity[onlyFor3DROI]
GLCM	GLCM_Homogeneity [=InverseDifference]	GLCM_Homogeneity [=InverseDifference]
	GLCM_Energy[=AngularSecondMoment]	GLCM_Energy[=AngularSecondMoment]
	GLCM_Contrast[=Variance]	GLCM_Contrast[=Variance]
	GLCM_Correlation	GLCM_Correlation
	GLCM_Entropy_log10	GLCM_Entropy_log10
	GLCM_Entropy_log2[=JointEntropy]	GLCM_Entropy_log2[=JointEntropy]
	GLCM_Dissimilarity	GLCM_Dissimilarity
GLRLM	GLRLM_SRE	GLRLM_SRE
	GLRLM_LRE	GLRLM_LRE
	GLRLM_LGRE	GLRLM_LGRE
	GLRLM_HGRE	GLRLM_HGRE
	GLRLM_SRLGE	GLRLM_SRLGE
	GLRLM_SRHGE	GLRLM_SRHGE
	GLRLM_LRLGE	GLRLM_LRLGE
	GLRLM_LRHGE	GLRLM_LRHGE
	GLRLM_GLNU	GLRLM_GLNU
	GLRLM_RLNU	GLRLM_RLNU
	GLRLM_RP	GLRLM_RP
NGLDM	NGLDM_Coarseness	NGLDM_Coarseness
	NGLDM_Contrast	NGLDM_Contrast
	NGLDM_Busyness	NGLDM_Busyness
GLZLM	GLZLM_SZE	GLZLM_SZE
	GLZLM_LZE	GLZLM_LZE
	GLZLM_LGZE	GLZLM_LGZE
	GLZLM_HGZE	GLZLM_HGZE
	GLZLM_SZLGE	GLZLM_SZLGE
	GLZLM_SZHGE	GLZLM_SZHGE
	GLZLM_LZLGE	GLZLM_LZLGE
	GLZLM_LZHGE	GLZLM_LZHGE
	GLZLM_GLNU	GLZLM_GLNU
	GLZLM_ZLNU	GLZLM_ZLNU
	GLZLM_ZP	GLZLM_ZP

**Table 2:** Clinical characteristics of patients.

Characteristics	Training Cohort *N* = 156		*p* Value	Testing Cohort *N* = 66		*p* Value
Pathological subtype	LUAD = 117	LUSC = 39		LUAD = 52	LUSC = 14	
age (mean ± SD)	62.42 ± 9.66	62.05 ± 8.55	0.833	60.04 ± 8.93	62.07 ± 11.01	0.475
sex (%)			1.000			0.724
male	71(60.7)	23(59.0)		36 (69.2)	11 (78.6)	
female	46 (39.3)	16 (41.0)		16 (30.8)	3 (21.4)	
Smoking Status			<0.001			0.003
Current or ever	58(49.6)	35(89.7)		19 (46.5)	12 (85.7)	
Non	59 (50.4)	4 (10.3)		33 (63.5)	2 (14.3)	
Tumor Location (%)			0.054			0.893
central	20 (17.1)	13 (33.3)		8 (15.4)	3 (21.4)	
peripheral	97 (82.9)	23 (66.7)		44 (84.6)	11 (78.6)	
Maximum diameter of tumor (%)			0.188			1.000
>3 cm	65 (55.6)	27 (69.2)		36 (73.1)	8 (71.4)	
<3 cm	52 (44.4)	12 (30.8)		16 (26.9)	6 (28.6)	
lymph node metastasis (%)			1.000			0.941
yes	36 (30.8)	12 (30.8)		12 (23.1)	4 (28.6)	
no	81 (69.2)	27 (69.2)		30 (76.9)	8 (71.4)	
T stage (%)			0.869			0.716
T1	36 (30.8)	13 (33.3)		7 (13.5)	3 (21.4)	
T2	56 (47.9)	20 (51.3)		38 (73.1)	8 (57.1)	
T3	11 (9.4)	3 (7.7)		5 (9.6)	2 (14.3)	
T4	14 (12.0)	3 (7.7)		2 (3.8)	1 (7.1)	
N stage (%)			0.770			0.782
N0	81 (69.2)	27 (69.2)		40 (76.9)	10 (71.4)	
N1	12 (10.3)	5 (12.8)		3 (5.8)	1 (7.1)	
N2	23 (19.7)	6 (15.4)		8 (15.4)	2 (14.3)	
N3	1 (0.9)	1 (2.6)		1 (1.9)	1 (7.1)	
M stage (%)			1.000			0.671
M0	107 (91.5)	36 (92.3)		49 (94.2)	12 (85.7)	
M1	10 (8.5)	3 (7.7)		3 (5.8)	2 (14.3)	
AJCC TNM stage (%)			0.623			0.502
I	63 (53.8)	19 (48.7)		29 (55.8)	8 (57.1)	
II	14 (12.0)	8 (20.5)		11 (21.2)	1 (7.1)	
III	30 (25.6)	9 (23.1)		9 (17.3)	3 (21.4)	
IV	10 (8.5)	3 (7.7)		3 (5.8)	2 (14.3)	
CYFRA21-1 (mean ± SD)	4.79 ± 2.91	7.19 ± 4.58	<0.001	5.62 ± 2.77	12.4 ± 5.20	0.003
Pro-GRP (mean ± SD)	49.76 ± 11.65	50.48 ± 13.57	0.749	50.55 ± 13.27	42.15 ± 10.42	0.032
SCCA (median [IQR])	1.30 (0.80, 84.49)	2.00 (1.15, 84.49)	0.362	49.29 (0.80, 84.49)	6.50 (3.88, 9.20)	0.750
CEA (median [IQR])	6.80 (3.10, 33.85)	4.20 (2.35, 33.85)	0.116	31.27 (3.00, 33.85)	4.15 (2.58, 6.05)	0.035
NSE (median [IQR])	11.75 (9.70, 11.80)	11.75 (11.40, 15.05)	0.009	11.75 (9.67, 11.75)	10.72 (2.90, 13.07)	0.532

CYFRA21-1 = cytokeratin 19 fragment antigen21-1, Pro-GRP = pro-gastrin-releasing peptide, SCCA = squamous cell carcinoma antigen, CEA = carcinoembryonic antigen, NSE = neuron-specific enolase.

**Table 3:** Prediction performance abilities of models in the testing cohorts.

Group	Model	Testing Cohort			
		AUC	ACC	Sen	Spe
Three models corresponding to λ.1se of Lasso regression	PET Primary model	0.823 (0.663–0.927)	0.818 (0.672–0.894)	0.786 (0.567–0.979)	0.750 (0.630–0.860)
	PET External model	0.830 (0.679–0.924)	0.820 (0.683–0.971)	0.786 (0.466–0.938)	0.808 (0.667–0.939)
	PET plus model	0.850 (0.684–0.939)	0.818 (0.697–0.893)	0.857 (0.549–1.000)	0.769 (0.654–0.873)
Four groups corresponding to λ.min of Lasso regression	CT plus model	0.804 (0.594–0.919)	0.818 (0.678–0.894)	0.714 (0.383–0.928)	0.865 (0.722–0.972)
	PET plus model	0.848 (0.702–0.935)	0.818 (0.703–0.873)	0.857 (0.652–1.000)	0.769 (0.639–0.848)
	PET/CT plus model	0.857 (0.649–0.955)	0.864 (0.727–0.939)	0.786 (0.443–0.938)	0.907 (0.691–0.917)
	PET/CT plus and clinical model	0.880 (0.697–0.979)	0.909 (0.818–0.984)	0.714 (0.389–0.906)	0.962 (0.829–1.000)

AUC area under the subject operating characteristic curve; ACC, accuracy; Sen, sensitivity; Spe, specificity.

**Table 4:** C-index of models for 1000 bootstrap and ten-fold cross-validation in the testing cohort.

	Model	1000-Bootstrap	Ten-Fold Cross-Validation
		C Index	Average C Index
Three models corresponding to λ.1se of Lasso regression	PET Primary model	0.813 (0.716–0.911)	0.847
	PET External model	0.799 (0.698–0.900)	0.823
	PET plus model	0.807 (0.705–0.910)	0.850
Four groups corresponding to λ.min of Lasso regression	CT plus model	0.730 (0.596–0.864)	0.804
	PET plus model	0.781 (0.670–0.892)	0.848
	PET/CT plus model	0.801 (0.692–0.909)	0.857
	PET/CT plus and clinical model	0.826 (0.721–0.930)	0.880

## Data Availability

The data that support the findings of this study are available on request from the corresponding author.

## Abbreviations

The following abbreviations are used in this manuscript:

PET/CT	Positron emission tomography/computed tomography
NSCLC	Non-small-cell lung cancer
LUAD	Lung adenocarcinoma
LUSC	Lung squamous cell carcinoma
Acc	Accuracy
Sen	Sensitivity
Spe	Specificity
ROC	Receiver operating curve
DCA	Decision curve analysis
AUC	Area under curve
CYFRA21-1	Cytokeratin fragment antigen21-1
Pro-GRP	Pro-gastrin-releasing peptide
SCCA	Squamous cell carcinoma antigen
CEA	Carcinoembryonic antigen
NSE	Neuron-specific enolase
